# Seed dormancy regulates germination response to smoke and temperature in a rhizomatous evergreen perennial

**DOI:** 10.1093/aobpla/ply042

**Published:** 2018-07-04

**Authors:** Hongyuan Ma, Todd E Erickson, David J Merritt

**Affiliations:** 1 Northeast Institute of Geography and Agroecology, Chinese Academy of Sciences, Shengbei Street, Changchun, Jilin, China; 2 Kings Park Science, Department of Biodiversity, Conservation and Attractions, Fraser Avenue, Kings Park, Western Australia, Australia; 3 School of Biological Sciences, The University of Western Australia, 35 Stirling Highway, Crawley, Western Australia, Australia

**Keywords:** Cold stratification, dormancy cycling, embryo growth, germination stimulant, secondary dormancy, temperature

## Abstract

Seed dormancy status regulates the response of seeds to environmental cues that can trigger germination. *Anigozanthos flavidus* (Haemodoraceae) produces seeds with morphophysiological dormancy (MPD) that are known to germinate in response to smoke, but embryo growth dynamics and germination traits in response to temperatures and after-ripening have not been well characterized. Seeds of *A. flavidus*, after-ripened for 28 months at 15 °C/15 % relative humidity, were incubated on water agar, water agar containing 1 μM karrikinolide (KAR_1_) or 50 μM glyceronitrile at 5, 10, 15, 20, 25, 20/10 and 25/15 °C for 28 days. After incubation at 5, 10 and 25 °C for 28 days, seeds were transferred to 15 °C for another 28 days. Embryo growth dynamics were tested at 5, 10, 15 and 25 °C. Results demonstrated that fresh seeds of *A. flavidus* had MPD and the physiological dormancy (PD) component could be broken by either glyceronitrile or dry after-ripening. After-ripened seeds germinated to ≥80 % at 15–20 °C while no additional benefit of germination was observed in the presence of the KAR_1_ or glyceronitrile. Embryo length significantly increased at 10 °C, and only slightly increased at 5 °C, while growth did not occur at 25 °C. When un-germinated seeds were moved from 5–10 °C to 15 °C for a further 28 days, germination increased from 0 to >80 % in significantly less time indicating that cold stratification may play a key role in the germination process during winter and early spring in *A. flavidus*. The lower germination (<50 %) of seeds moved from 25 to 15 °C was produced by the induction of secondary dormancy. Induction of secondary dormancy in seeds exposed to warm stratification, a first report for *Anigozanthos* species, suggests that cycling of PD may be an important mechanism of controlling germination timing in the field.

## Teaser

Fresh seeds of *A. flavidus* have differentiated but underdeveloped embryos and possess non-deep morphophysiological dormancy (MPD). The MPD could be broken by smoke-derived chemical of glyceronitrile. However, after-ripened seeds (dry stored at 15 °C for 28 months) could germinate readily at temperatures 15–20 °C while glyceronitrile did not improve germination percent or speed following loss of physiological dormancy (PD). Embryo growth could occur at 5 and 10 °C, faster with increasing temperature, but could not germinate until the temperature was 15 °C. Warm temperature above 25 °C induced secondary dormancy in seeds and no embryo elongation.

## Introduction

Seed germination is the initial and most crucial stage in the life cycle of most flowering plants. Seeds of the majority of plant species are dormant at maturity (Baskin and Baskin 2014). Dormancy is an adaptive trait that helps regulate the timing of seed germination ensuring that the chances of seedling survival are greatest. Seeds respond to a host of environmental factors to control germination timing including fluctuation in moisture availability, temperature, light, and cues provided by naturally occurring chemicals such as nitrates, and compounds present in smoke (Baker *et al.* 2005a; Downes *et al.* 2014; Abu *et al.* 2016; Villa-Reyes and Barrera 2016; Cox *et al.* 2017).

Five primary kinds (classes) of seed dormancy are recognized, i.e. physical dormancy (PY), physiological dormancy (PD), morphological dormancy (MD), morphophysiological dormancy (MPD) and combinational dormancy (PY+PD), with PD being the most common (Baskin and Baskin 2004). Seeds with non-deep PD, the least stringent form of PD control, commonly cycle between dormant and non-dormant states in the soil seed bank in response to seasonal changes in environmental conditions (Baskin and Baskin 2004; Chauhan *et al.* 2006; Long *et al.* 2011).

Since seed dormancy status regulates the response of seeds to environmental cues that can trigger germination (Flematti *et al.* 2011; Abdelgadir *et al.* 2013; Florentine *et al.* 2016), unravelling the interplay between dormancy status and the efficacy of germination cues is important in resolving the seed germination ecology of species in their natural environments (Tieu *et al.* 2001a, b; Baker *et al.* 2005a, b; Merritt *et al.* 2007; Hidayati *et al.* 2012) and for managing seeds stored under *ex situ* conditions (Merritt and Dixon 2011; Erickson *et al.* 2017).

For seeds of the species-rich flora of southwestern Australia, primary processes involved in dormancy loss include dry after-ripening (i.e. dormancy loss during warm, dry conditions such as those that seeds experience in summer), warm stratification (warm, moist conditions such as those seeds experience in autumn) and wet/dry cycling (conditions common during the sporadic rainfall of early autumn) (Turner *et al.* 2006, 2009; Merritt *et al.* 2007; Hidayati *et al.* 2012). Temperatures of 25–35 °C commonly promote dormancy loss, with seeds then germinating in winter (the period of reliable rainfall) upon the onset of cooler temperatures of 15–20 °C (Bellairs and Bell 1990; Merritt *et al.* 2007). Dormancy loss through cold stratification is not often reported, although it is known to occur in some species such as *Xyris lanata* (Merritt *et al.* 2007).

In fire-prone ecosystems, fire plays a major role in the recruitment of plant species via seed (Dixon *et al.* 1995; Merritt *et al.* 2006; Turner *et al.* 2009). Seedlings emerge en masse from the soil seed bank in the winter growing season immediately following a fire, with seed germination being promoted by chemicals in smoke that are deposited into the soil during the fire (Flematti *et al.* 2013). Chemicals present in smoke, including karrikins (KAR_1_) and glyceronitrile, along with crude smoke products (aerosol smoke and smoke water) promote the germination of at least 1200 Australian species (Dixon *et al.* 2009; Flematti *et al.* 2013, 2015). While likely to produce the same ecological response in the field, the effect of the compounds is species specific, with seeds of some species responding only to KAR_1_ but not glyceronitrile and vice versa (Flematti *et al.* 2011; Erickson *et al.* 2017; Lewandrowski *et al.* 2018).

Recent studies have highlighted the complexities of the germination response to smoke and that seed receptivity to smoke-derived chemicals is strongly regulated by seed dormancy status (Merritt *et al.* 2007), prior-hydration history (Long *et al.* 2010) and even seed age (Liyanage and Ooi 2017). For example, in seeds with PD, seed sensitivity to smoke can cycle seasonally as the depth of dormancy varies in response to changing soil temperature and moisture conditions (Baker *et al.* 2005b; Long *et al.* 2011). Other seeds appear receptive to smoke regardless of the previous conditions experienced (Merritt *et al.* 2007), and it has been proposed that seeds can be classified according to whether their response to smoke is inherent or inducible (Long *et al.* 2011). Thus, to understand the ecology of seed germination in fire-prone floras, studies are required on the role of dormancy-breaking factors in regulating the response to smoke and also on the germination requirements and characteristics of non-dormant seeds and how these determine the expression of the smoke response.

The genus *Anigozanthos* is a member of the ancient Gondwanan family Haemodoraceae which consists of highly divergent and relictual taxa in southwest Western Australia (Hopper *et al.* 2009; Ayre *et al.* 2017). *Anigozanthos* species produce seeds with MPD. Seeds with MPD have an underdeveloped embryo that must grow inside the seed prior to germination and physiological blocks to germination (i.e. PD) that must also be overcome (Baskin and Baskin 2005; Baskin *et al.* 2015). Dormancy release and germination of a number of *Anigozanthos* species have long been known to be associated with fire-related cues including heat and smoke (Dixon *et al.* 1995; Tieu *et al.* 2001a, b). More recently, it has been shown that seed germination of *Anigozanthos manglesii*, *A. humilis* and *A. viridis* is promoted by glyceronitrile (Flematti *et al.* 2011; Downes *et al.* 2014), a smoke-derived chemical that, in the presence of water, hydrolyzes to release cyanide (Flematti *et al.* 2011). Seeds of a few *Anigozanthos* species after-ripen in laboratory storage, with the germination percentage of a seed population exposed to smoke or glyceronitrile becoming progressively higher during after-ripening and germination in water also increasing, albeit to a lesser extent (Tieu *et al.* 2001c; Downes *et al.* 2014). This response indicates that as dormancy is released there is a widening of the conditions suitable for germination. However, in seeds of several *Anigozanthos* species, the germination potential varies substantially within and between species in response to dormancy alleviation pretreatments, exposure to a suite of germination stimulants and the post-harvest handling and storage conditions (Tieu *et al.* 2001c; Merritt *et al.* 2007; Downes *et al.* 2014). Seeds of *Anigozanthos flavidus* provide one such example (Flematti *et al.* 2011; Downes *et al.* 2013, 2014; Phillips *et al.* 2014).

*Anigozanthos flavidus* is distributed along the most southwestern parts of Western Australia and forms large, densely clumped plants to 3 m tall. This species produces numerous yellow-green flowers with seeds maturing in early autumn (March), and these seeds will drop off from the mother plant after maturity. To date, research on seeds of *A. flavidus* has focussed on the progression of dormancy loss during soil burial, the effects of after-ripening in the laboratory on dormancy break and the germination response of dormant seeds to smoke-derived products. For example, two recent studies on the germination traits of *A. flavidus* seeds found that freshly collected seeds were dormant at dispersal (0 % germination), and following a period of dry after-ripening in the laboratory (e.g. 10 weeks at 35 °C, or 3–3.5 years at 22 °C) (Downes *et al.* 2013, 2014). They became responsive to smoke water or glyceronitrile with germination increasing from 0 to ca. 55 % (Downes *et al.* 2013, 2014). In contrast, another recent study of *A. flavidus* seeds sourced from 13 populations found that freshly collected seeds were dormant (average germination 32 %) but highly responsive to glyceronitrile (average germination 86 %) (Phillips *et al.* 2014).

Although dry after-ripening is thought to be the predominant means of dormancy alleviation in seeds of *Anigozanthos* spp., the response of the seeds to cold or warm stratification has not been tested. In a study by Downes *et al.* (2014), after-ripening resulted in >50 % germination of smoke/glyceronitrile-treated *A. flavidus* seeds, but seeds buried for 1 year in the soil and retrieved in autumn germinated to only 17 % following smoke or glyceronitrile treatment. A similar discrepancy between after-ripened and soil-buried seeds was reported by Tieu *et al.* (2001c) for seeds of *A. manglesii*. These results suggest that more complex interactions beyond after-ripening may be influencing dormancy status and germination in the soil environment. In particular, suppression of germination of seeds buried in soil compared to laboratory after-ripened seeds and the apparent variation in dormancy depth across different populations suggest that induction of secondary dormancy is possible under unfavourable conditions.

In this study, we examined the germination traits of *A. flavidus* seeds aiming to determine whether the (i) incubation temperatures and warm and/or cold stratification will influence the seed germination potential by affecting the elongation of embryos; (ii) seed sensitivity to the smoke-derived products glyceronitrile and/or KAR_1_ is dictated by dormancy status.

## Methods

### Seeds

Seeds of *A. flavidus* were collected in late March 2011 from 50 plants in a natural population near Northcliffe in southwest Western Australia (34°38′32″S, 116°07′25″E), cleaned in the laboratory and then pooled. The annual average temperature and annual precipitation of the Northcliffe area is 15 °C and 1158 mm, respectively. From November to April, the monthly maximum and minimum temperatures are 25–30 °C and 14–18 °C, respectively (http://www.bom.gov.au/climate/averages/tables/cw_009034_All.shtml). Precipitation predominantly falls during winter from June to August (ca. 50 % of total precipitation), with ca. 6 % occurring in summer (from December to February). Cleaned seeds were allowed to after-ripen in a controlled environment room at 15 °C and 15 % RH for 28 months, until germination experiments commenced in July 2013. Mean (±SE) length and mass of individual seeds were 2.39 ± 0.01 mm and 0.65 ± 0.02 mg, respectively. The mass of seeds was determined for seeds stored at 15 °C and 15 % RH. The viability of seeds (four replicates of 100 seeds) was tested via x-ray analysis (Faxitron MX-20 Digital X-ray Cabinet, Tucson, AZ, USA). Final viability was deemed to be 99–100 % due to the presence of uniformly, white/grey x-ray images of seeds and well-developed endosperm.

### Germination of freshly collected seeds

To confirm the presence and the initial effect of dormancy on seeds of *A. flavidus*, freshly collected seeds were incubated in Petri dishes containing 0.7 % (w/v) water agar (Gelita Australia Pty Ltd) or water agar containing 50 μM glyceronitrile, and assessed for germination at constant 15 °C on a 12/12 h light/dark regime in July 2011. Prior to germination testing, seeds were sterilized in a 2 % (w/v) calcium hypochlorite (Ca(OCl)_2_) solution for 30 min and rinsed twice in sterilized distilled water.

### Effects of temperature, KAR_1_ and glyceronitrile on germination of after-ripened seeds

Seeds from the same accession described above, after-ripened for 28 months, were incubated in Petri dishes containing either: 0.7 % (w/v) water agar, water agar containing 1 μM karrikinolide (KAR_1_) or water agar containing 50 μM glyceronitrile. Glyceronitrile and KAR_1_ were synthesized in pure form following Flematti *et al.* (2011) and Flematti *et al.* (2004), respectively. Seeds were incubated at 5, 10, 15, 20, 25, 20/10 or 25/15 °C with a daily 12 h photoperiod of 30 μmol m^−2^ s^−1^ 400–700 nm, cool white fluorescent light in growth chambers (Contherm BIOSYN 6000CP, Hutt City, New Zealand). For the alternating temperatures, the high temperature was implemented during the 12-h light period. Seeds were considered to be germinated upon radicle emergence, and germination was scored every 2–3 days for 28 days. Four dishes of 25 seeds were used for each treatment.

### Movement of seeds to 15 °C

After incubating seeds at 5, 10 and 25 °C for 28 days, no germination was observed. Therefore, all the Petri dishes containing non-germinated seeds were moved to the 15 °C incubator. Subsequent germination was then scored at 2- to 3-day intervals for another 28 days.

### Effects of temperature on embryo growth

To observe the rate of embryo growth, four replicates of 25 seeds were incubated on water agar, or 50 μM glyceronitrile at 5, 10, 15 and 25 °C and embryo length was determined by dissecting seeds and measuring the embryo and seed lengths under a binocular microscope equipped with an ocular micrometre. For seeds at 15 °C, the embryo length was determined after 0, 3, 6, 9, 12 and 15 days of incubation, and for seeds at 5, 10 and 25 °C embryo length was determined after 28 days of incubation.

### Data analysis

Generalized linear models (GLMs) with a binomial error structure and logit link function were used to compare the proportional data for the final germination of *A. flavidus* for the temperatures and smoke-related chemical treatments. The time to obtain 50 % germination (*T*_50_) was calculated using the equation (Farooq *et al.* 2005):

$$T_{50}=t_{i}+\frac{\left(\frac{N}{2}-N_{i}\right)(t_{j}-t_{i})}{\left(N_{j}-N_{i}\right)}$$

where *N* is the final germinated seed number, *N*_*j*_ and *N*_*i*_ are the cumulative number of seeds germinated by adjacent counts at times *t*_*j*_ and *t*_*i*_, respectively, when *N*_*i*_ < *N*/2 < *N*_*j*_. We used a one-way or two-way analysis of variance (ANOVA; *P* < 0.05) to compare *T*_50_ and embryo length (mm) data with temperatures and smoke chemicals as factors. Tukey’s test was used for multiple comparisons when the final germination among treatments was significant. All analyses were carried out using the R statistical platform (R Core Development Team 2014).

## Results

### Germination of fresh seeds

**Figure 1.**
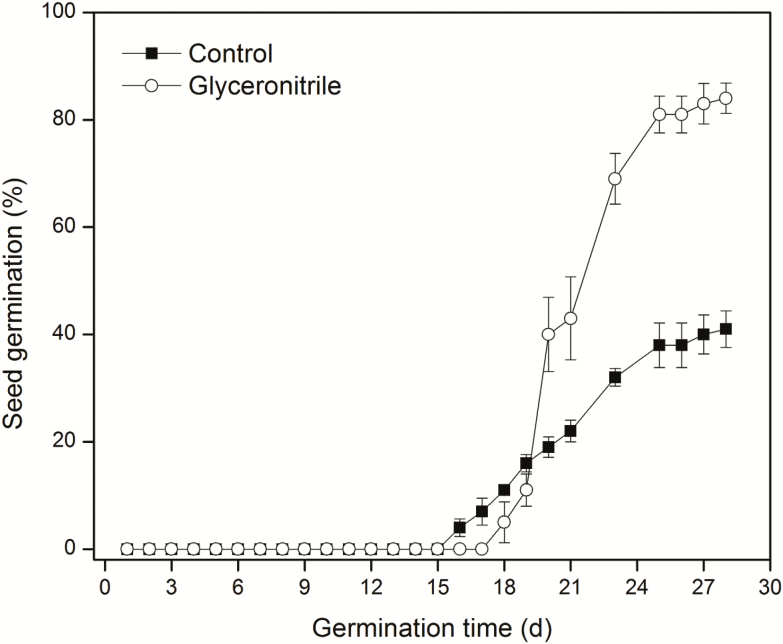
Germination dynamics (mean % ± SE) of fresh *Anigozanthos flavidus* seeds incubated at 15 °C in a 12/12 h light/dark regime during 28-day incubation in Petri dishes containing 0.7 % (w/v) water agar (control, black square) or water agar containing 50 μM glyceronitrile (white circle). Four dishes of 25 seeds were used for each treatment.

Most freshly collected seeds were dormant with a germination percentage of 41 ± 3.4, but dormancy could be broken by the application of glyceronitrile with a germination percentage of 84 ± 2.8 (Fig. 1). Significant differences were obtained between germination of the control and the glyceronitrile-treated seeds (*P* < 0.0001).

### Effects of temperature, KAR_1_ and glyceronitrile on germination of after-ripened seeds

**Figure 2.**
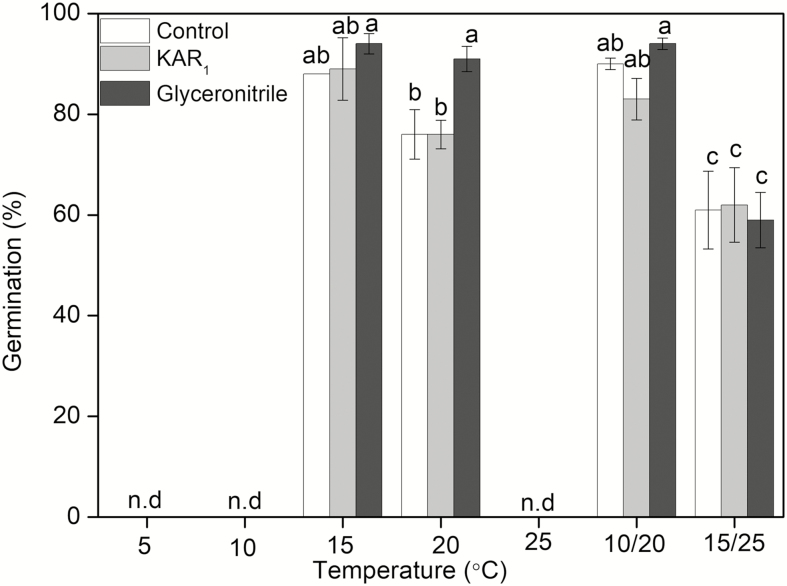
Germination (mean % ± SE) of after-ripened *Anigozanthos flavidus* seeds incubated at 5, 10, 15, 20, 25, 20/10 and 25/15 °C in a 12/12 h light/dark regime for 28 days on water (control), KAR_1_ or glyceronitrile. Different letters indicate significant differences (*P* < 0.05) between germination after 28 days across all treatments at different incubation temperatures. ‘n.d.’ means no germination was recorded at these temperatures.

For the after-ripened seeds, high germination was obtained under the intermediate temperature regimes of 15, 20 and 20/10 °C. A significant reduction in germination was evident at 25/15 °C while no germination was observed at 5, 10 and 25 °C (Fig. 2). Seed germination of *A. flavidus* was significantly affected by the incubation temperature (df = 3, χ^2^ = 100.902, *P* < 0.0001) and smoke stimulants (df = 2, χ^2^ = 7.242, *P* = 0.0268), but the interaction of germination stimulants × incubation temperature did not significantly affect seed germination (df = 6, χ^2^ = 11.914, *P* < 0.064).

### Effects of temperature, KAR_1_ and glyceronitrile on germination speed

Germination rate was significantly faster at 20 °C and slowest at 25/15 °C (Table 1) and temperature strongly affected *T*_50_ (*F*_3, 12_ = 57.187, *P* < 0.001). However, neither smoke chemicals (*F*_2, 9_ = 1.098, *P* = 0.344) nor the interaction of chemicals × temperature (*F*_6, 36_ = 0.607, *P* = 0.723) had a significant effect on germination rate.

**Table 1.** Time to obtain 50 % germination (*T*_50_) of after-ripened *Anigozanthos flavidus* seeds incubated at 5, 10, 15, 20, 25, 20/10 and 25/15 °C and movement from 5, 10, 25 to 15 °C in a 12/12 h light/dark regime on water (control), KAR_1_ or glyceronitrile. Data in the table represent mean ± SE. No germination was observed at 5, 10 and 25 °C and their *T*_50_ are expressed as /. The 5→15, 10→15 and 25→15 represent seeds were moved from 5, 10 and 25 °C to 15 °C, respectively.

Temperature (°C)	5	10	15	20	25	10/20	15/25	5→15	10→15	25→15
H_2_O	/	/	16.4 ± 0.2	13.6 ± 0.1	/	16.8 ± 0.3	17.7 ± 0.8	10.2 ± 0.4	2.6 ± 0.2	18.4 ± 0.7
KAR_1_	/	/	16.4 ± 0.2	13.7 ± 0.1	/	17.5 ± 0.3	17.9 ± 0.7	10.4 ± 0.4	2.8 ± 0.1	18.0 ± 0.7
Glyceronitrile	/	/	16.2 ± 0.2	13.9 ± 0.1	/	17.3 ± 0.3	18.8 ± 0.7	10.3 ± 0.3	3.2 ± 0.3	17.7 ± 0.7

### Movement of seeds to 15 °C

**Figure 3.**
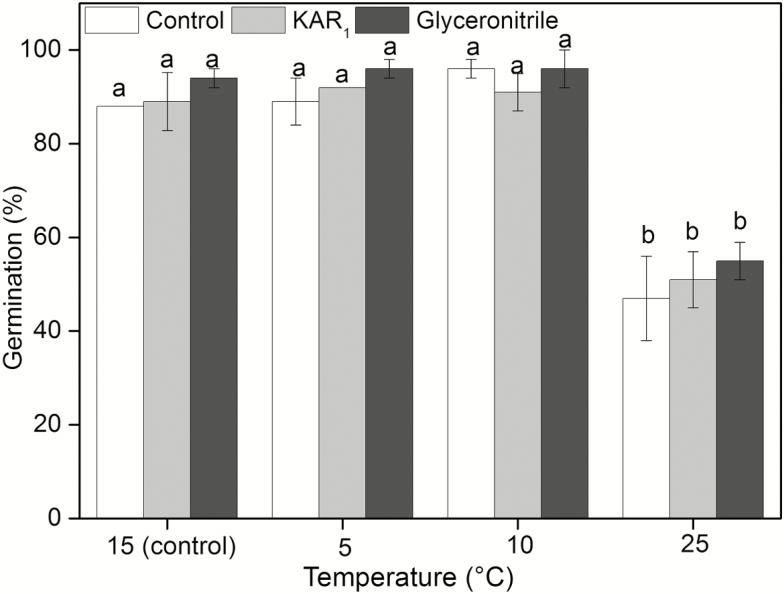
Mean germination (% ± SE) of after-ripened *Anigozanthos flavidus* seeds cold stratified (5 °C, 10 °C) or warm stratified (25 °C) for 4 weeks in water, KAR_1_ or glyceronitrile and then moved to 15 °C for an additional 4 weeks with seed germination at 15 °C as control. Before movement, no germination occurred at 5, 10 and 25 °C. Different letters indicate significant differences (*P* < 0.05) between mean germination time across all treatments.

Before movement, no seeds germinated at 5, 10 and 25 °C. After moving seeds to the 15 °C incubator, seeds formerly incubated at 5 and 10 °C reached similar germination as the control (15 °C), but significantly lower germination was obtained for the seeds formerly incubated at 25 °C (Fig. 3). Temperatures from which seeds were removed significantly affected germination (df = 2, χ^2^ = 200.623, *P* < 0.0001). However, smoke chemicals (df = 2, χ^2^ = 2.345, *P* = 0.310) and the interaction of smoke chemicals × temperature (df = 4, χ^2^ = 4.309, *P* = 0.366) had no significant effects on germination.

The *T*_50_ of seeds moved from 10 °C was 2.6–3.2 days which was significantly less than that moved from 5 °C (10.2–10.4 days) and 25 °C (17.7–18.4 days) (Table 1). Temperature significantly affected *T*_50_ (*F*_2, 9_ = 734.192, *P* < 0.001). However, neither smoke chemicals (*F*_2, 9_ = 0.000, *P* = 1.000) or their interaction with temperature (*F*_4, 27_ = 0.419, *P* = 0.793) had a significant effect on germination.

### Effects of incubation temperature on embryo growth

**Figure 4.**
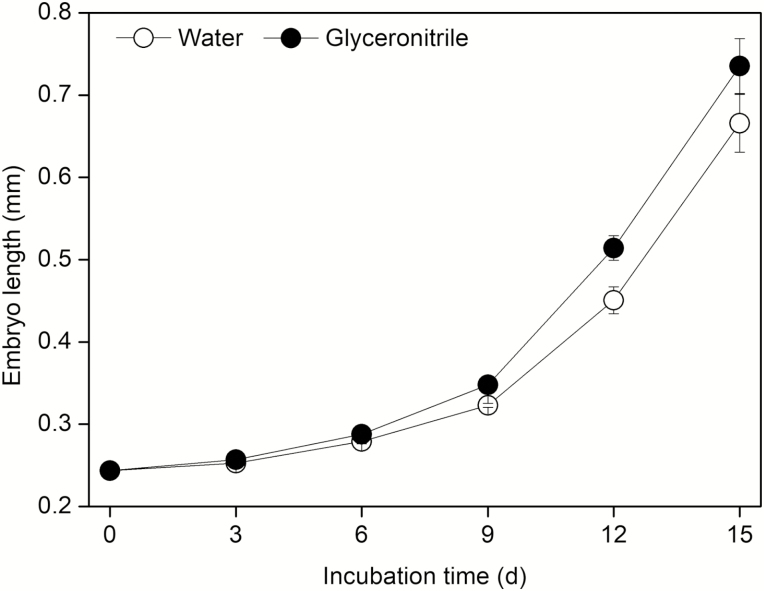
Embryo growth (mean ± SE) dynamics of after-ripened *Anigozanthos flavidus* seeds incubated at 15 °C in Petri dishes containing 0.7 % (w/v) on water agar (control, white circle) or water agar containing 50 μM glyceronitrile (black circle). The embryo length was determined after 0, 3, 6, 9, 12 and 15 days of incubation. Four dishes of 100 seeds were used for each sampling time point.

**Figure 5.**
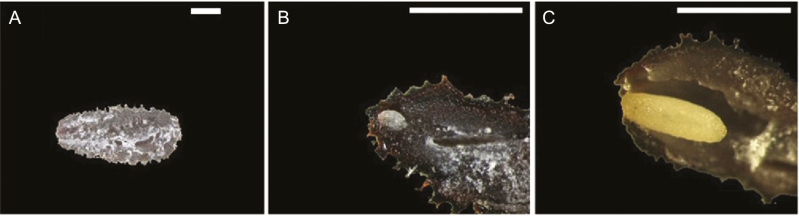
Embryo growth for after-ripened seeds of *Anigozanthos flavidus* in this study was demonstrated by continually measuring the increase in length (mm) of embryos during incubation at suitable temperatures (see Fig. 4). Examples of an intact seed (A), embryo before growth (B) and embryo before germination (C) are shown visually here. Scale bar = 1 mm.

At 15 °C, embryos grew slowly but measurably within 9 days of incubation and then increased exponentially from 9 to 15 days when some radicles began to protrude through the seed coat (Figs 4 and 5). The incubation time significantly inhibited the elongation of embryos (*F*_5, 18_ = 90.549, *P* < 0.001), while there was no significant difference between the glyceronitrile and the control (H_2_O) treatments (*F*_1, 6_ = 4.157, *P* = 0.097).

**Figure 6.**
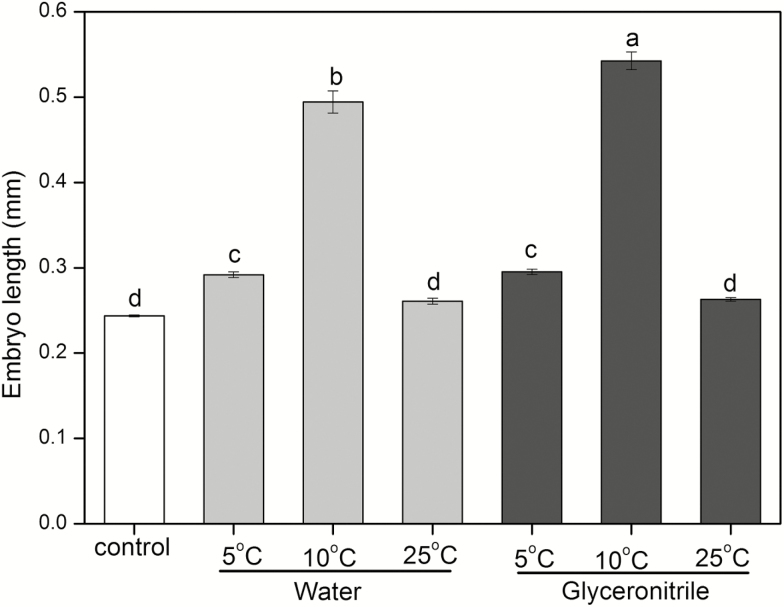
Embryo length (mean ± SE) of after-ripened *Anigozanthos flavidus* seeds incubated at 5, 10 and 15 °C for 28 days on filter paper moistened with water or glyceronitrile. Control represents mean embryo length of seeds prior incubation. Bars with different letters are significantly different at *P* < 0.05.

Seeds incubated at 5 and 10 °C exhibited significant embryo growth, but there were no growth observed in seeds incubated at 25 °C (Fig. 6). The embryo length of seeds incubated in glyceronitrile was significantly longer than that of seeds in water at 10 °C, but no differences were recorded at the other temperatures. The ANOVA analysis showed that temperature had the greatest effect on embryo growth (*F*_2, 9_ = 112.368, *P* < 0.01), and there were no significant effects of the presence/absence of glyceronitrile (*F*_1, 6_ = 1.380, *P* = 0.06). There was also a significant interaction between temperature and the presence/absence of glyceronitrile on embryo growth (*F*_2, 18_ = 6.722, *P* < 0.01).

## Discussion

It is well recognized that temperature regulates both seed dormancy break and germination worldwide (Baskin and Baskin 2004; Finch-Savage and Leubner-Metzger 2006; Liyanage and Ooi 2017). Seeds may be dormant at maturity, and then come out of, or go back into, dormancy in response to the moisture and temperature of their environment (Kebreab and Murdoch 1999; Baskin and Baskin 2006; Vandelook *et al.* 2008; Scholten *et al.* 2009; Baskin and Baskin 2014; Liyanage *et al.* 2017). In this study, we showed that the freshly collected seeds of *A. flavidus* have MPD and the PD component could be overcome by either exposure to glyceronitrile, a smoke-derived germination stimulant previously shown to promote germination more consistently in seeds of the Haemodoraceae (Flematti *et al.* 2011), or by dry after-ripening (e.g. in this study storage at 15 °C/15 % RH for up to 28 months was utilized).

Following the dispersal of the *A. flavidus* seeds at the end of March or April, when autumn temperatures are cooling and early season rains commence, PD is most likely alleviated under natural conditions due to a combination of dry after-ripening and periods of stratification when soils are periodically wet (Merritt *et al.* 2007). The loss of PD in seeds of *Anigozanthos* species, through mechanisms such as dry after-ripening and exposure to fire-related cues, has long been recognized (Tieu *et al.* 2001c; Flematti *et al.* 2011; Downes *et al.* 2013, 2014; Phillips *et al.* 2014).

Unique to this study, however, is the previously unreported benefit of cold stratification of *A. flavidus* seeds in the promotion of embryo growth and germination at temperatures aligned to the winter season. For some Mediterranean climate species, seeds germinate over a wide range of temperatures (e.g. 5–30 °C), once non-dormant (Zaidi *et al.* 2010; Martínez-García *et al.* 2012; Copete *et al.* 2014; Veiga-Barbosa and Perez-Garcia 2014; Martínez-Baniela *et al.* 2016; Cross *et al.* 2017). However, temperatures at which maximum germination occurs usually correspond with seasonal conditions that are most favourable for the growth and survival of seedlings (Bellairs and Bell 1990; Mackenzie *et al.* 2016), and seeds of *Anigozanthos* spp. have consistently been observed to germinate at a narrow temperature range of 15–20 °C, including in this study (Tieu *et al.* 2001c; Flematti *et al.* 2011; Downes *et al.* 2013, 2014).

Unsurprisingly, embryo growth also was centred around these optimum germination temperatures. For instance, we found that once PD was alleviated through dry storage, growth of the underdeveloped embryo occurred on moist substrates at ca. 15 °C (Figs 4 and 5). Embryo length significantly increased at 10 °C, but did not protrude the seed coat, and only slightly increased at 5 °C. Embryo growth did not occur at 25 °C, meaning there is strict temperature regulation, aligned to cooler winter season conditions, with respect to initiation of embryo growth. Interestingly, when non-germinated seeds were moved from 5–10 °C to 15 °C for a further 28 days, germination increased from 0 to >80 % in significantly less time (i.e. time to 50 % germination reduced from ca. 13–16 days down to ≤3 days). This indicates that cold stratification may play a key role in the germination process during winter and early spring in *A. flavidus*, whereby embryo growth commences at temperatures <15 °C but is not completed until a shift into the optimum temperature range of 15–20 °C is achieved. This response to simulated seasonal temperatures of southwest Western Australia suggests that germination would be aligned to late winter and early spring when temperature increases are in unison with high soil moisture conditions (*sensu*Fig. 5 of Merritt *et al.* 2007).

Although warm stratification breaks dormancy in many species with MPD (Baskin and Baskin 2004), we found the opposite in seeds of *A. flavidus*, whereby seeds exposed to extended periods of warm (25 °C), moist conditions lost the ability to germinate and appeared to enter into secondary dormancy; a first report for the *Anigozanthos* genus. The presence of secondary dormancy is supported by the lack of germination occurring at 25 °C, and 40–50 % germination occurring after shifting seeds from 25 to 15 °C. Further, the embryos of non-germinated *A. flavidus* seeds under these conditions were assessed for viability post-germination testing and were confirmed to be firm, white, and therefore, presumed viable. It should be noted that our assessment of dormant viable embryos from non-germinated *A. flavidus* seeds was based on a cut test, which has been previously identified as a robust method for dormant species in Australia (Ooi *et al.* 2004). Induction of non-dormant seeds into secondary dormancy (i.e. MD→MPD) by warm stratification is not uncommon. For instance, it has been reported in seeds of *Delphinium fissum* subsp. *sordidum* from the Iberian Peninsula, southwestern Europe (Herranz *et al.* 2010). Further, secondary dormancy in *Narcissus alcaracensis* seeds from southern Spain, induced by high temperature, could be broken by certain cold stratification treatments (Herranz *et al.* 2017). These examples of cycling between a non-dormant state and secondary dormancy, induced by warmer temperatures, indicate that seed germination of *A. flavidus* is synchronized with the beginning of winter.

In unison with dormancy cycling, fire-related germination cues have also been shown to assist in field recruitment of many Mediterranean species (Dixon *et al.* 1995). The most commonly applied fire-related chemicals stimulating seed germination are karrikinolide (KAR_1_) (Flematti *et al.* 2004, 2005; Daws *et al.* 2007; Stevens *et al.* 2007) and glyceronitrile (Flematti *et al.* 2011; Downes *et al.* 2013; Phillips *et al.* 2014). These germination stimulants are widely used to study the influence of fire on seed dormancy and plant recruitment patterns in both conservation and restoration programs (Phillips *et al.* 2014; Commander *et al.* 2017; Cross *et al.* 2017; Erickson *et al.* 2017; Lewandrowski *et al.* 2018). Changes of seeds in sensitivity to smoke with alleviation of seed dormancy have been reported in several species (Brown and van Staden 1997; Baker *et al.* 2005b; Merritt *et al.* 2007). Seeds of *A. manglesii* become more smoke responsive with increasing shelf storage time (Tieu *et al.* 2001c). Seed germination of *Actinotus leucocephalus* (Apiaceae) gradually increases with the combination of increasing laboratory storage time and smoke water treatment (Baker *et al.* 2005b). Significant promotion of seed germination by glyceronitrile in *A. flavidus* (Fig. 1) has been previously reported in freshly collected (Phillips *et al.* 2014) and after-ripened seeds (Downes *et al.* 2013, 2014). In our current study, glyceronitrile slightly promoted germination of after-ripened *A. flavidus* seeds but not significantly. As shown in Figs 4 and 6, significant interaction between temperature and glyceronitrile on embryo growth was observed. This demonstrated that glyceronitrile promoted embryo growth only at certain temperatures. Therefore, it is likely that the dormancy status of the seeds, and hence their response to some of the germination stimulating treatments, changed as a result of dry storage at 15 °C (after-ripening) for 28 months.

## Conclusions

Fresh seeds of *A. flavidus* possess a type of non-deep MPD that could be broken by smoke-derived chemical glyceronitrile. However, after-ripened seeds of *A. flavidus* germinated readily at temperatures 15–20 °C and neither glyceronitrile nor KAR_1_ improved germination percentage or speed following loss of PD. Embryo growth could occur at 5 and 10 °C but seeds could not germinate until the temperature is about 15 °C in winter when precipitation is also suitable for germination and establishment of *A. flavidus*. Temperatures above 25 °C induced secondary PD in seeds and no embryo elongation occurred. The dormancy cycling of MPD↔MD→ND↔PD in *A. flavidus* seeds may be an important mechanism of controlling germination timing in the field.

## Sources of Funding

This work was supported by the National Natural Science Foundation of China (41771058, 41371260), the National Basic Research Program of China (2015CB150802) and the National Key Research & Development Program of China (2016YFC0501200) and the National Key Basic Survey of Resources (2015FY110500).

## Contributions by the Authors

H.M., T.E.E. and D.J.M. designed the experiments. H.M. conducted the experiments and analysed the data. H.M., T.E.E. and D.J.M. interpreted the data. H.M., T.E.E. and D.J.M. wrote the manuscript.

## Conflict of Interest

None declared.
